# Heparan sulfate binding protein treatment ameliorates neuropathology and behavioral abnormalities in mucopolysaccharidosis IIIB mice

**DOI:** 10.1038/s41420-025-02648-w

**Published:** 2025-08-01

**Authors:** Serenella Anzilotti, Melania Scarcella, Mariangela Ciampa, Noemi Di Muraglia, Camilla Anastasio, Chiara Fiorentino, Federica Rossin, Luigi Avallone, Giuseppe Pignataro, Luigi Michele Pavone, Valeria De Pasquale

**Affiliations:** 1 Department of Human Sciences and Quality of Life Promotion, San Raffaele University, Rome, Italy; 2 Department of Molecular Medicine and Medical Biotechnology, Medical School, University of Naples Federico II, Naples, Italy; 3 Division of Pharmacology, Department of Neuroscience, Reproductive and Dentistry Sciences, Medical School, University of Naples Federico II, Naples, Italy; 4 Department of Precision Medicine, University of Campania Luigi Vanvitelli, Naples, Italy; 5 Department of Veterinary Medicine and Animal Production, University of Naples Federico II, Naples, Italy

**Keywords:** Neurodegeneration, Neurodegeneration

## Abstract

Mucopolysaccharidosis IIIB (MPS IIIB) is a metabolic neurodegenerative disorder caused by a deficiency of the lysosomal enzyme α-N-acetylglucosaminidase (NAGLU), which is involved in the degradation of heparan sulfate (HS). Affected patients exhibit progressive neurodegeneration, behavioral disturbances, and a shortened lifespan. Currently, there is no effective treatment for MPS IIIB. We have recently developed a new therapeutic strategy based on the use of the HS-binding protein NK1, a spliced variant of hepatocyte growth factor. Here, we demonstrate that treating *Naglu*^−/−^ mice with recombinant NK1 ameliorates neuropathology by reducing HS storage, lysosomal dysfunction, autophagy imbalance, and neuroinflammation in the cortex and hippocampus of MPS IIIB mouse brains. Furthermore, we found that recombinant NK1 treatment improves cognitive behavior and motor activity in *Naglu*^−/−^ mice, as assessed using open field, object recognition, and T-maze tests. Our findings suggest that recombinant NK1 is a promising candidate for the treatment of MPS IIIB and other lysosomal storage diseases associated with central nervous system dysfunction.

## Introduction

Mucopolysaccharidosis IIIB (MPS IIIB), also referred to as Sanfilippo syndrome type B, is a neurometabolic disease classified as a lysosomal storage disorder (LSD). It results from mutations in the gene encoding α-N-acetylglucosaminidase (NAGLU), one of the lysosomal enzymes responsible for the breakdown of heparan sulfate (HS) [1–7]. A deficiency in the NAGLU enzyme results in the incomplete degradation and accumulation of HS within the lysosomes and on cell membranes throughout the body, leading to tissue and organ damage [6]. In addition to peripheral manifestations such as organomegaly, cardiac and respiratory insufficiency, bone and joint problems, hearing loss, and retinopathy, the central nervous system (CNS) is significantly affected in MPS IIIB, resulting in distinctive neuropathological changes [7]. Individuals with MPS IIIB exhibit progressive neurodegeneration accompanied by severe behavioral dysregulation, hyperactivity, co-occurring autism, sleep disturbances, intellectual disabilities, motor dysfunction, and a reduced lifespan [8, 9].

Studies in animal models of MPS IIIB and other MPS types with CNS involvement have demonstrated that the neuropathology associated with the disease entails the dysregulation of multiple cellular processes, leading to neuroinflammation characterized by substantial astrocytic and microglial activation, synaptic disorganization, impaired autophagy, mitochondrial defects, oxidative stress, altered cellular signaling, and abnormalities in additional pathways [6, 10–14]. Notably, lysosomal compartment size and HS levels progressively increase over time across all regions of the MPS IIIB mouse brains [11, 12]. Lysosomal enlargement and substrate accumulation correlate with a significant degree of secondary storage of gangliosides, neuronal damage, and cell death, as well as the upregulation of pro-inflammatory factor expression, alterations in synaptic proteins, dysregulation of MAPK signaling, and other neurodegenerative processes [11, 12, 15,16]. Furthermore, MPS IIIB mice exhibit behavioral abnormalities starting at 4 months of age, which progressively worsen with age [11, 17–19], effectively recapitulating the human disease phenotype. The MPS IIIB mouse model has provided critical insights into understanding the neuropathogenic events driving the progressive neurodegeneration and behavioral issues in MPS IIIB. It has also been extensively utilized to evaluate numerous therapeutic approaches for the disease [20, 21]. However, despite significant efforts, no effective disease-modifying treatment has been developed or approved to date for MPS IIIB [22].

Given the urgent need to identify therapies that can either cure or significantly alleviate the symptoms of this severely debilitating disease, as well as other MPS types that accumulate pathogenic HS fragments and exhibit a similar CNS phenotype, we have recently developed a novel therapeutic approach utilizing an HS binding protein: the recombinant hepatocyte growth factor/scatter factor (HGF/SF) natural spliced variant, NK1 [16, 23–25]. Our previous in vitro studies have demonstrated that recombinant NK1 effectively reduces HS accumulation and lysosomal pathology in MPS IIIB patient-derived fibroblasts, along with a neuronal cellular model of the disease established by *NAGLU* gene expression silencing in the human neuroblastoma cell line SK-NBE [16, 26, 27]. Notably, our investigations into the mechanisms of action of the recombinant NK1 protein have revealed its ability to reverse deregulated cell signaling in cellular models of MPS IIIB, and treatment with recombinant NK1 induced the differentiation of *NAGLU*-silenced SK-NBE into neuron-like cells. Furthermore, we discovered that NK1 treatment rescues the disease phenotype by modulating autophagy pathways. Additionally, a targeted metabolomic approach combined with Seahorse analysis of the oxygen consumption rate profile has demonstrated that NK1 treatment rescues impaired mitochondrial functions in both the *NAGLU*-silenced neuroblastoma cell line and the brains of *Naglu* knockout (*Naglu*^−/−^) mice [27].

Encouraged by the promising in vitro results in MPS IIIB cellular models, we investigated the in vivo effects of recombinant NK1 treatment on brain neuropathology and behavioral changes in a mouse model of MPS IIIB. Our study showed that recombinant NK1 treatment decreases lysosome and HS accumulation in the brains of *Naglu*^−/−^ mice. Additionally, administering recombinant NK1 significantly affected autophagy, neuroinflammation, and both cognitive and motor behaviors in MPS IIIB mice. The findings from this study highlight the need for further research to explore the potential therapeutic effectiveness of recombinant NK1 for treating MPS IIIB and other LDSs with a neurodegenerative phenotype.

## Results

### NK1 treatment reduces lysosomes and HS in the brains of *Naglu*^−/−^ mice

**Fig. 1:**
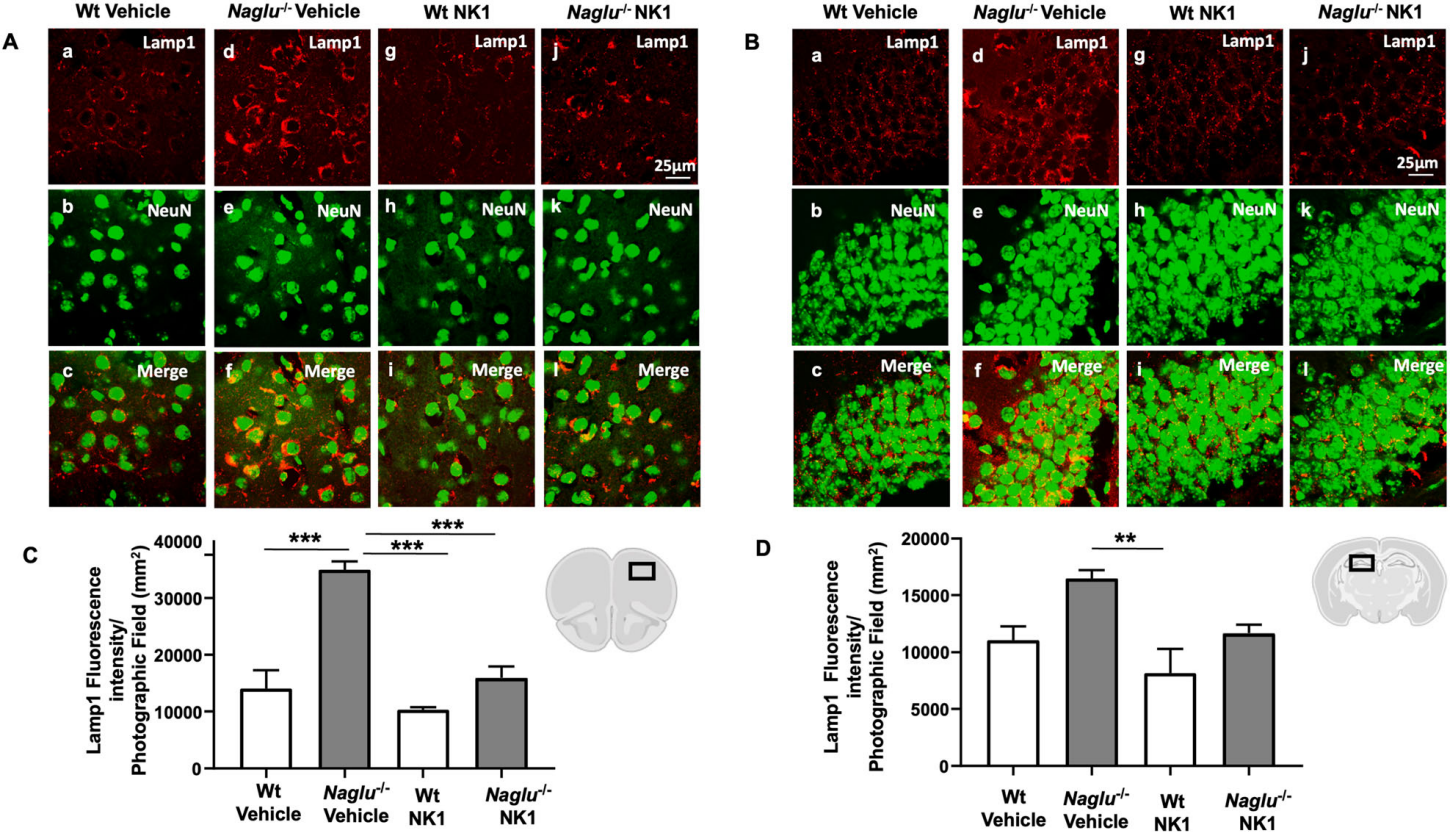
Effect of NK1 treatment on Lamp1 fluorescence intensity. Double labeling of Lamp1 and NeuN in cortex (**A**) and hippocampus (CA1) (**B**) of Wt mice + vehicle (a–c), *Naglu*^−/−^ + vehicle (d–f), Wt + NK1 (g–i) and *Naglu*^−/−^ + NK1 (j–l). **C** Lamp1 fluorescence intensity (mm^2^) in the mouse cortex. **D** Lamp1 fluorescence intensity (mm^2^) in the mouse hippocampus. Scale bar 25 µm. Statistically significant differences among means were determined by one-way ANOVA followed by Tukey’s correction for multiple comparisons test: **p < 0.01, ***p < 0.001.

In MPS IIIB disease, the lack of NAGLU activity leads to the accumulation of lysosomes and HS in the tissues and organs of affected individuals [16, 26, 27]. To evaluate the in vivo effect of recombinant NK1 on lysosomal accumulation in the brains of *Naglu*^−/−^ mice, Lamp1 (lysosomal associated membrane protein 1) immunostaining was performed in the prefrontal cortex and hippocampus of brains isolated from four groups of mice (Fig. 1). Colocalization analysis was conducted using an antibody against NeuN, a neuron-specific nuclear protein that is stably expressed in most postmitotic neurons of the vertebrate nervous system [28]. Colocalization of Lamp1 and NeuN was visualized in neuronal cells of *Naglu*^−/−^ mice (Fig. 1Af, Al, Bf, Bl). NK1 treatment did not affect NeuN and Lamp1 fluorescence staining in either the cortex or hippocampus of both treated and untreated Wt mice (Fig. 1Ac, Ai, Bc, Bi). In contrast, an intense Lamp1 immunoreaction was detected in the prefrontal cortex and hippocampus of the vehicle-treated *Naglu*^−/−^ group of mice (Fig. 1Ad, Bd) as compared to Wt mice (Fig. 1Aa, Ba). Notably, Lamp1 immunofluorescence was attenuated in the NK1-treated *Naglu*^−/−^ group (Fig. 1Aj, Bj). Quantification of Lamp1 fluorescence intensity showed a significant decrease in both the cortex and hippocampus of *Naglu*^−/−^ mice chronically treated with recombinant NK1 compared to vehicle-treated *Naglu*^−/−^ mice (Fig. 1C, D). These results demonstrate that NK1 treatment significantly reduces lysosomal accumulation in the brains of MPS IIIB mice.

**Fig. 2:**
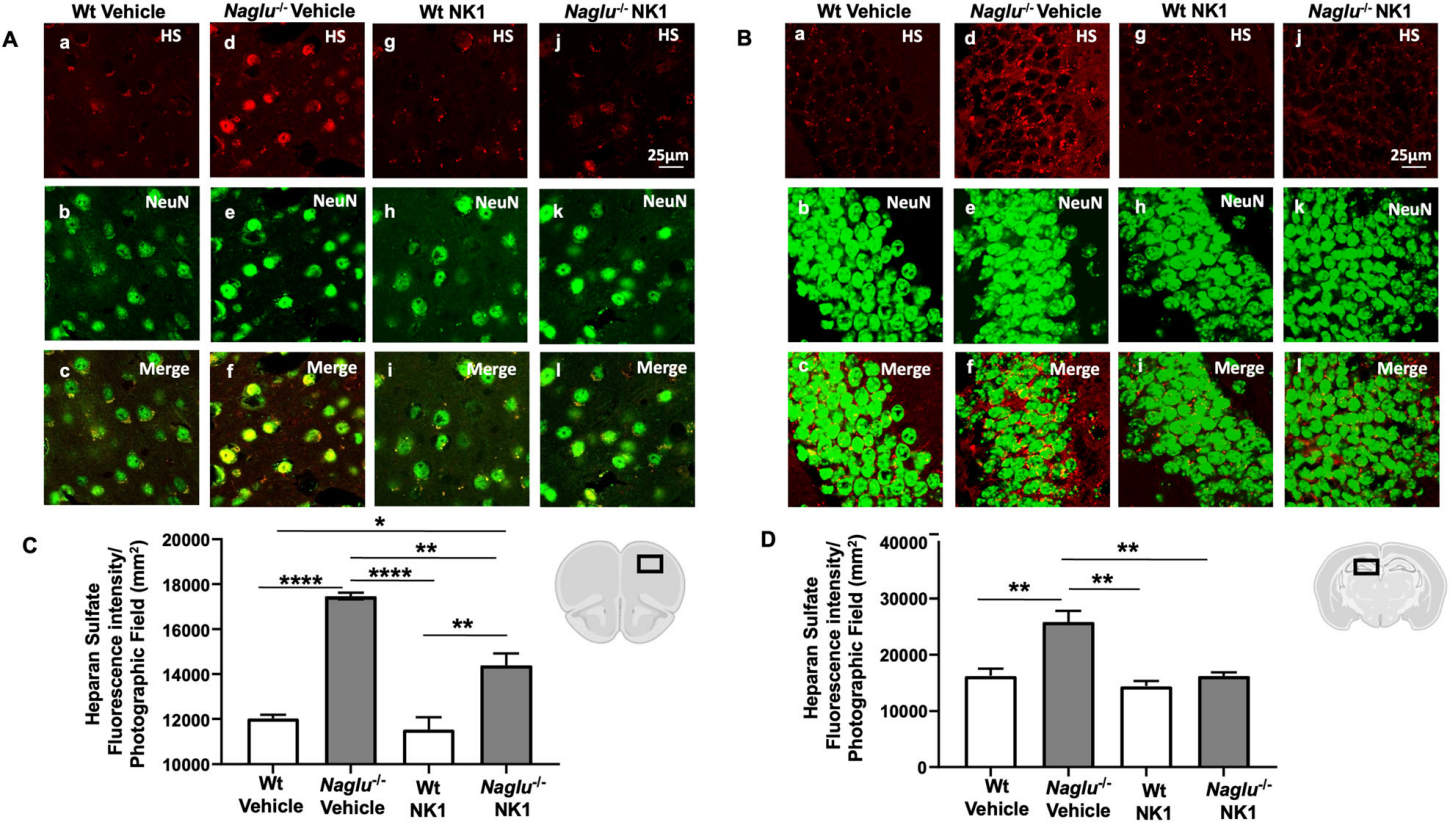
Effect of NK1 treatment on HS fluorescence intensity. Double labeling of HS and NeuN in cortex (**A**) and hippocampus (CA1) (**B**) of Wt mice + vehicle (a–c), *Naglu*^−/−^ + vehicle (d–f), Wt + NK1 (g–i) and *Naglu*^−/−^ + NK1 (j–l). **C** HS fluorescence intensity (mm^2^) in the mouse cortex. **D** HS fluorescence intensity (mm^2^) in the mouse hippocampus. Scale bar 25 µm. Statistically significant differences among means were determined by one-way ANOVA followed by Tukey’s correction for multiple comparisons test: *p < 0.05, **p < 0.01, ***p < 0.001, ****p < 0.0001.

Thus, we evaluated the effect of recombinant NK1 treatment on HS in the *Naglu*^−/−^ mouse brain. To this end, HS and NeuN immunostaining were conducted in the prefrontal cortex and hippocampus isolated from the brain tissues of the four groups of mice (Fig. 2). Recombinant NK1 did not affect HS fluorescence staining in either the cortex or the hippocampus of both treated and untreated Wt mice (Fig. 2Ac, Ai, Bc, Bi). In contrast, an intense HS immunoreaction was detected in the prefrontal cortex and hippocampus of the vehicle-treated *Naglu*^−/−^ group of mice (Fig. 2Ad, Bd) compared to Wt mice (Fig. 2Aa, Ba). Notably, HS immunofluorescence was attenuated in the NK1-treated *Naglu*^−/−^ group of mice (Fig. 2Aj, Bj). Interestingly, the HS signal was present in both nuclear and cytosolic areas of the cortical neurons of vehicle-treated *Naglu*^−/−^ mice (Fig. 2Ad), while in NK1-treated animals, the signal appeared punctate and localized in the cytosol of the cells, similar to Wt animals (Fig. 2Aagj). However, in the hippocampus, the HS signal was only punctate and present in the cytosol across all experimental groups (Fig. 2B).

Indeed, the quantification of HS fluorescence intensity revealed a significant decrease in the cortex and hippocampus of *Naglu*^−/−^ mice chronically treated with recombinant NK1, compared to vehicle-treated *Naglu*^−/−^ mice (Fig. 2C, D).

The observed enhancement of Lamp1 and HS fluorescence intensity due to abnormal lysosomal substrate accumulation in the brain tissues of control (vehicle-treated) *Naglu*^−/−^ mice, compared to the Wt group of mice, correlates well with previous reports by us and other authors, both in cellular models in vitro and in the MPS IIIB mouse model in vivo. These studies demonstrate an enlargement of the lysosomal compartment and increased HS storage in diseased cells and brain tissues [12, 26, 27, 29, 30]. The findings of this in vivo investigation indicate that NK1 treatment significantly reduces substrate storage and lysosomal pathology in the brains of MPS IIIB mice, which can have important effects on the regulation of lysosomal functions in mouse neurons.

### NK1 treatment reduces autophagy imbalance in the brains of *Naglu*^−/−^ mice

**Fig. 3:**
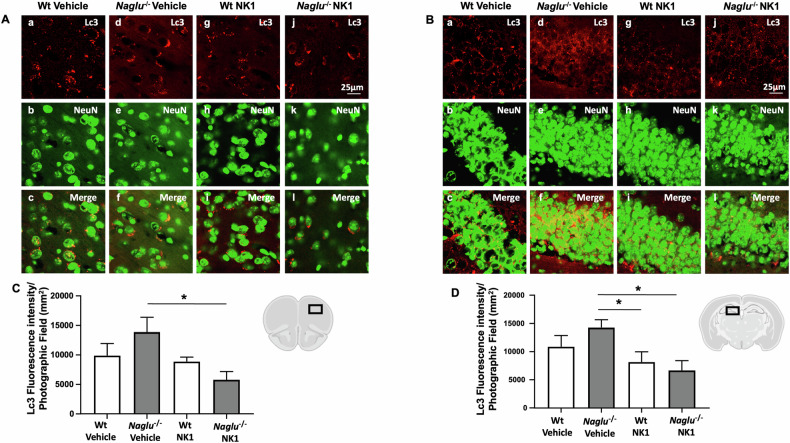
Effect of NK1 treatment on Lc3 fluorescence intensity. Double labeling of Lc3 and NeuN in cortex (**A**) and hippocampus (CA1) (**B**) of Wt mice + vehicle (a–c), *Naglu*^−/−^ + vehicle (d–f), Wt + NK1 (g–i) and *Naglu*^−/−^ + NK1 (j–l). **C** Lc3 fluorescence intensity (mm^2^) in the mouse cortex. **D** Lc3 fluorescence intensity (mm^2^) in the mouse hippocampus. Scale bar 25 µm. Statistically significant differences among means were determined by one-way ANOVA followed by Tukey’s correction for multiple comparisons test: *p < 0.05.

To determine whether the observed efficacy of recombinant NK1 in reducing lysosomes and substrate storage in the MPS IIIB mouse model would impact autophagy in mouse brain tissues, this study evaluated the autophagosome marker protein Lc3 in the neurons of the prefrontal cortex and hippocampus of both vehicle- and NK1-treated Wt and *Naglu*^−/−^ mice. Fluorescent microscopy was carried out by using a rabbit polyclonal anti-Lc3 antibody that recognizes both Lc3-I form and the lipidated Lc3-II isoform [31, 32]. The members of Lc3 protein family commonly exhibit a homogeneous cytosolic distribution that changes into puncta upon induction of autophagy as they conjugate with autophagosomal membranes. Indeed, elevated levels of Lc3 are typically associated with an increased number of autophagosomes, which may result from either enhanced autophagosome synthesis, reduced autophagosome turnover (impaired fusion of autophagosomes with lysosomes), or both. In both the cortex and hippocampus of mouse brains, Lc3 fluorescence appeared punctate and localized in the cytosol of neurons across all mouse groups, although with varying intensity levels (Fig. 3A, B). In brain tissues from *Naglu*^−/−^ mice, Lc3 labeling intensity was significantly higher than in samples from the other animal groups and was notably reduced after NK1 treatment (Fig. 3C, D). Recombinant NK1 administration did not affect Lc3 fluorescence in the control (Wt) group of mice.

These results demonstrate the capability of NK1 to interfere with autophagosome accumulation in the neurons of the *Naglu*^−/−^ mouse brain, and they are consistent with our previous findings that recombinant NK1 can modulate autophagy in in vitro cellular models of MPS IIIB disease [33–36].

### NK1 treatment reduces neuroinflammation in the brain of *Naglu*^−/−^ mice

**Fig. 4:**
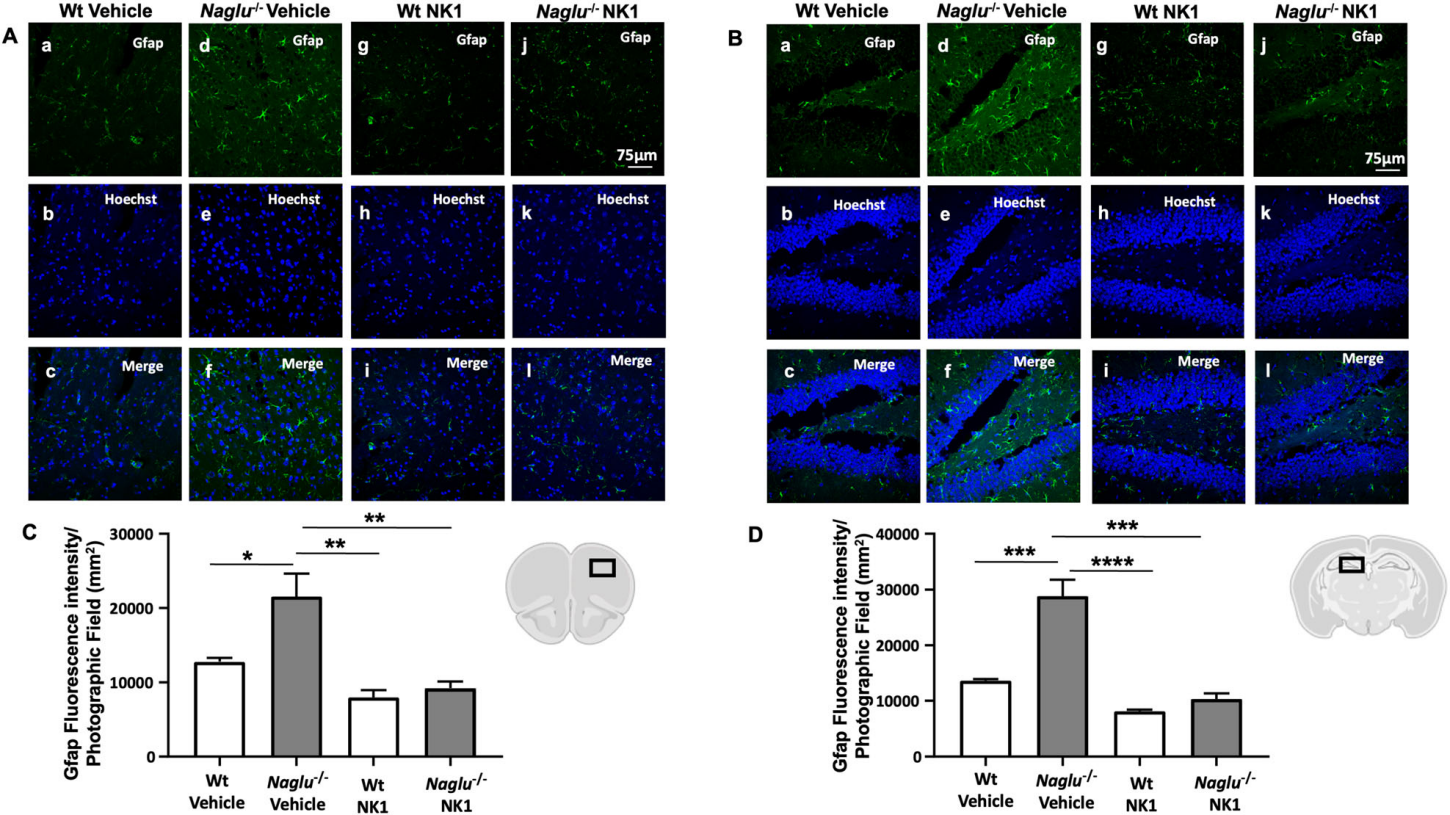
Effect of NK1 treatment on Gfap fluorescence intensity. Double labeling of Gfap and Hoechst in cortex (**A**) and hippocampus (CA1) (**B**) of Wt mice + vehicle (a–c), *Naglu*^−/−^ + vehicle (d–f), Wt + NK1 (g–i) and *Naglu*^−/−^ + NK1 (j–l). **C** Gfap fluorescence intensity (mm^2^) in the mouse cortex. **D** Gfap fluorescence intensity (mm^2^) in the mouse hippocampus. Scale bar 75 µm. Statistically significant differences among means were determined by one-way ANOVA followed by Tukey’s correction for multiple comparisons test: *p < 0.05, **p < 0.01, ***p < 0.001, ****p < 0.0001.

To investigate whether the reduction of substrate storage by NK1 treatment in *Naglu*^−/−^ mice leads to a decrease in neuroinflammation hallmarks in the brains of treated mice, we examined the effect of recombinant NK1 on astrogliosis and microgliosis. For this purpose, we performed immunofluorescence analysis using anti-Gfap (glial fibrillary acidic protein) antibodies as an astrocyte marker, and anti-Iba1 (ionized calcium-binding adaptor molecule 1) antibodies as a microglia marker in the prefrontal cortex and hippocampus of both vehicle- and NK1-treated Wt and *Naglu*^−/−^ mice. Scattered astrocyte cells were visualized by Gfap immunostaining in the cortex and hippocampus of Wt mice (Fig. 4Aa, Ag, Ba, Bg). In contrast, an intense astroglial reaction was evident in the vehicle-treated *Naglu*^−/−^ group (Fig. 4Ad, Bd), which was significantly attenuated in NK1-treated *Naglu*^−/−^ mice (Fig. 4Aj, Bj). Furthermore, fluorescence intensity quantification of Gfap staining showed a significant reduction in the cortex and hippocampus of NK1-treated *Naglu*^−/−^ mouse brains (Fig. 4C, D).

**Fig. 5:**
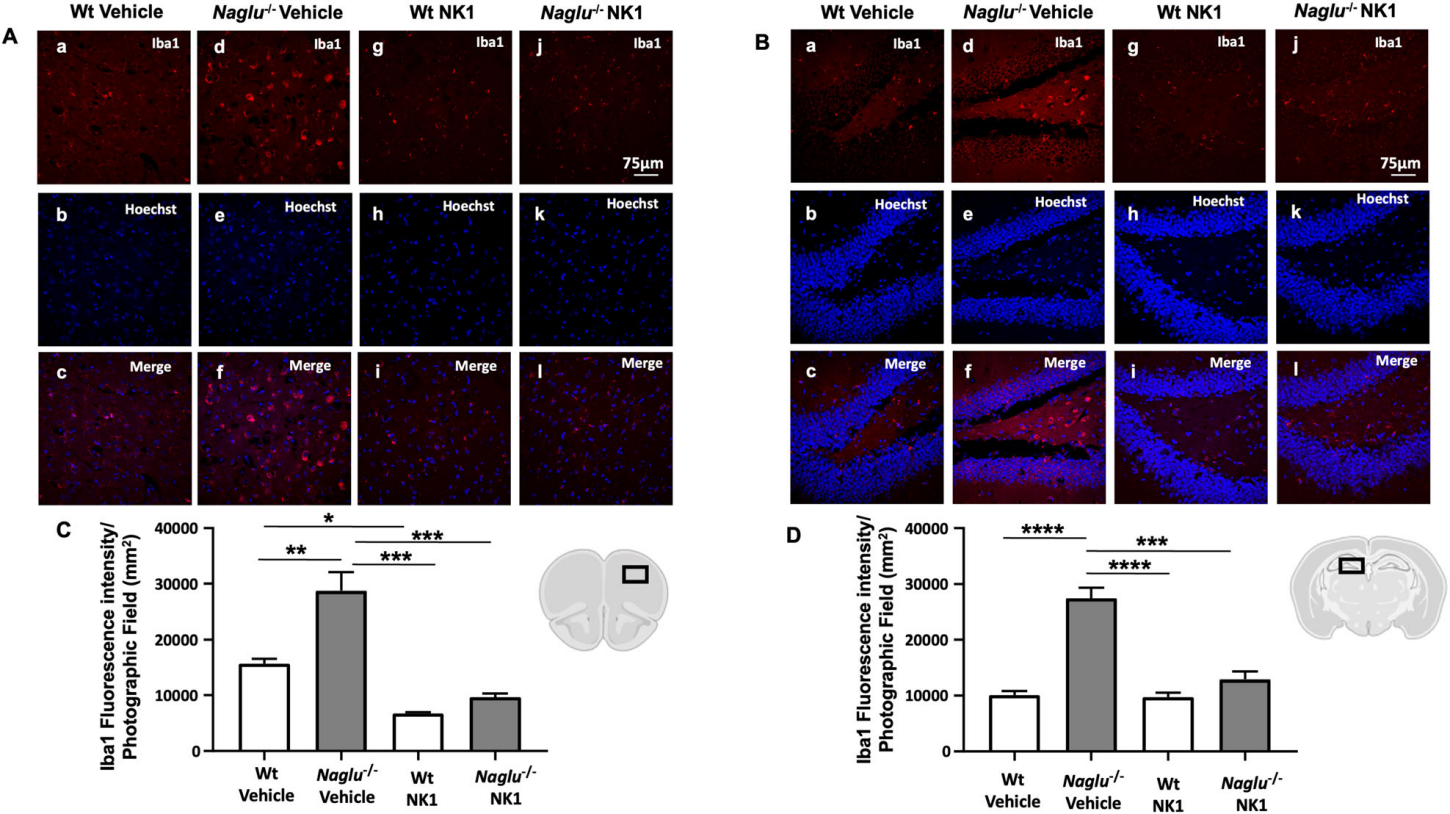
Effect of NK1 treatment on Iba1 fluorescence intensity. Double labeling of Iba1 and Hoechst in cortex (**A**) and hippocampus (CA1) (**B**) of Wt mice + vehicle (a–c), *Naglu*^−/−^ + vehicle (d–f), Wt + NK1 (g–i) and *Naglu*^−/−^ + NK1 (j–l). **C** Iba1 fluorescence intensity (mm^2^) in the mouse cortex. **D** Iba1 fluorescence intensity (mm^2^) in the mouse hippocampus. Scale bar 75 µm. Statistically significant differences among means were determined by one-way ANOVA followed by Tukey’s correction for multiple comparisons test: *p < 0.05, **p < 0.01, ***p < 0.001, ****p < 0.0001.

Furthermore, a strong activation of Iba1 immunostaining was evident in both the cortex and hippocampus of vehicle-treated *Naglu*^−/−^ mice compared to the Wt group (Fig. 5Aa, Ad, Ba, Bd). While NK1 treatment did not change Iba1 reactivity in the brain tissues of Wt mice (Fig. 5Ag, Bg), microglial activation was reduced in the brain tissues of NK1-treated *Naglu*^−/−^ mice (Fig. 5Aj, Bj). Indeed, quantification of Iba1 fluorescence intensity was significantly reduced in the cortex and hippocampus of chronic NK1-treated *Naglu*^−/−^ mice compared to vehicle-treated *Naglu*^−/−^ mice (Fig. 5C, D).

These results, while confirming previous evidence of astrocytosis and microglia activation in the MPS IIIB mouse model [11, 12], demonstrate the effectiveness of NK1 treatment in reducing this activation in the brains of *Naglu*^−/−^ mice. This suggests a beneficial effect of recombinant protein administration on the neuroinflammation affecting MPS IIIB patients.

### NK1 treatment improves cognitive behavior and motor activity of MPS IIIB mice

To investigate the effects of recombinant NK1 treatment on the spatial and recognition memory of MPS IIIB mice, a series of cognitive tests were conducted, including the open field test, object recognition test, and T-maze spontaneous alternation test.

**Fig. 6:**
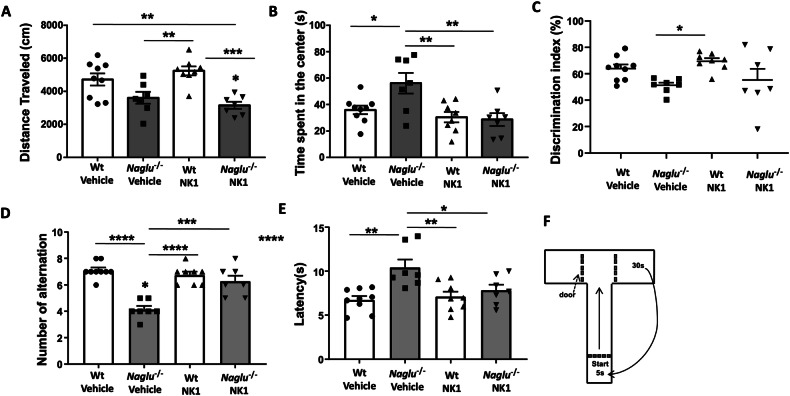
Effects of NK1 treatment on motor function and cognitive behavior. **A** distance traveled in open field cage (cm), **B** time spent in the center of the cage expressed in seconds, **C** discrimination index in object recognition test expressed in percentage, **D** Number of alternations in Tmaze test, **E** Latency to fall expressed in seconds. **F** Experimental protocol of Tmaze test. Statistically significant differences among means were determined by one-way ANOVA followed by Tukey’s correction for multiple comparisons test: *p < 0.05, **p < 0.01, ***p < 0.001, ****p < 0.0001.

The open field test was conducted to evaluate motor activity, measured as total distance traveled, and anxiety, indicated by time spent in the center of the cage. In this experiment, *Naglu*^−/−^ mice, regardless of treatment, exhibited reduced motor activity compared to Wt groups, as shown by total distance traveled (Fig. 6A). However, a significant effect of NK1 treatment was observed on the time spent in the center of the open field (Fig. 6B). Indeed, while vehicle-treated *Naglu*^−/−^ mice recorded a median time of 56.1 s, NK1-treated mice demonstrated a reduced time of ~28.7 s, similar to the Wt groups (Fig. 6B), indicating a more normative fear response to the open field environment with NK1 treatment. Additionally, no effects were observed with NK1 treatment in Wt mice (Fig. 6A, B).

The object recognition test was conducted to evaluate recognition memory and response to a novel environment. Mice were placed in an open field cage with two objects to explore while a video tracking system recorded their activity. During an initial training session, two identical objects were presented to the mice, and the time they spent exploring them was measured. Twenty-four hours after the removal of the objects, the mice were tested again, providing one object they had interacted with during training (familiar) and a new one (novel). The discrimination index decreased in *Naglu*^−/−^ mice treated with vehicle, which spent significantly less time exploring the novel object compared to Wt mice groups (Fig. 6C). This indicates reduced memory of the familiar object encountered during the training session. In contrast, the discrimination index increased in *Naglu*^−/−^ mice treated with recombinant NK1. Notably, three out of seven mice displayed the same behavior as the Wt group animals, as shown in Fig. 6C.

Finally, mice were challenged with a T-shaped maze to assess repetitive behavior and spatial working memory. The test consisted of ten successive trials, where mice spent 5 s in the starting box, had a maximum latency of 60 s to decide whether to proceed to the right or left arm of the T-maze, and were confined to the chosen arm for 30 s (Fig. 6F). As shown in Fig. 6D, vehicle-treated *Naglu*^−/−^ mice exhibited fewer alternations between the two arms of the maze compared to the Wt animals, indicating a higher rate of repetitive behavior and suggesting an impairment of spatial memory. After administering recombinant NK1, the number of alternations increased by 20%; indeed, over 10 repetitions of the T test, the mice alternated between the right and left arms 6 times (Fig. 6D). Finally, latency time also decreased in NK1-treated *Naglu*^−/−^ mice, demonstrating that these mice were faster in arm selection during the test than vehicle-treated mice (Fig. 6E).

## Discussion

Until now, no effective therapies have been identified for MPS III subtypes, including MPS IIIB, and treatments remain limited to supportive and palliative care to address the various multisystem issues associated with the disease. Several therapeutic approaches have been explored, including enzyme replacement therapy (ERT), substrate reduction therapy (SRT), hematopoietic stem cell transplants (HSCT), and gene therapy. However, these strategies have not yet produced an approved or accessible disease-modifying treatment for individuals suffering from MPS III [20, 21, 37, 38]. The unwanted side effects arising from the immune responses of patients undergoing cell and gene therapy [39–41], along with the fact that some of these therapeutic strategies require invasive routes of administration (i.e., intrathecal and intracerebroventricular injections), prolonged treatment times, and elevated costs for drug production and/or patient hospitalization, highlight the challenges in this field. Therefore, the search for effective drugs that can be delivered with reduced risk and minimal patient burden for treating neuropathic disorders associated with MPS IIIB and other MPS subtypes continues to represent an unmet medical need [42].

We recently developed a new therapeutic strategy utilizing the recombinant NK1 protein, which binds with high affinity to the undegraded HS accumulated on the surface and in the extracellular environment of cells in patients affected by MPS IIIB. This approach has proven effective in in vitro studies using cellular models of MPS IIIB, where the administration of NK1 reduced substrate storage and related lysosomal dysfunctions [16, 26, 27, 43]. In this in vivo study, we demonstrate for the first time that recombinant NK1 reduces neuropathology in the brains of MPS IIIB mice and also attenuates neurobehavioral deficits. While treatment with recombinant NK1 showed no toxic effects on animal health, we observed a significant reduction in substrate (HS) and lysosomal accumulation in the prefrontal cortex and hippocampus of MPS IIIB mice treated with NK1 for six months. The in vivo efficacy of recombinant NK1 in reducing HS storage and lysosomal defects in the brains of *Naglu*^−/−^ mice is significant considering that the primary accumulation of HS and related lysosomal dysfunctions are thought to be responsible for the pathological changes occurring in the CNS structures of MPS III models, such as neuroinflammation, autophagy impairment, mitochondrial defects, and alterations in cell signaling, among others [6, 10, 12, 44].

Although extensive research is needed to fully understand the molecular mechanism of action of NK1, the results of this in vivo study, together with those obtained in in vitro cellular models of the MPS IIIB disease, allow us to hypothesize with sufficient confidence that the binding of recombinant NK1 to the HS side chains of proteoglycans present on the cell surface and extracellular matrix significantly affect the regulatory functions and mechanisms that HS proteoglycans exert in multiple cellular pathways. Indeed, in MPS I, II, III, and VII subtypes, the accumulation of HS due to the enzymatic deficiency does not occur only in the lysosomes but also in other intracellular compartments and in the extracellular mileu as well [6, 26, 27]. In particular, post-mortem analysis has revealed HS accumulation at non-lysosomal sites in the brain of patients affected by the above MPS subtypes [30]. In addition, upregulation of membrane-bound and extracellular HS moieties has been detected in the brain of MPS IIIB mouse model [33]. Interestingly, in these MPS subtypes, the excess of HS in compartments other than lysosomes has been associated with the alterations of various cellular processes, including receptor activation by ligands, receptor responses, intracellular trafficking, and others [6, 10–12, 16, 26, 33, 34, 44]. On this basis, we previously demonstrated that recombinant NK1 treatment in MPS IIIB fibroblasts restores cellular signaling pathways disrupted in MPS IIIB disease consequently causing the reduction of HS storage and rescue of lysosomal defects. Additionally, we showed that exposure to recombinant NK1 reactivates physiological lysosome trafficking and exocytosis in *NAGLU*-silenced human neuroblastoma cells, SK-NBE [26]. Therefore, considering the established role of HS proteoglycans in the development and homeostasis of CNS, along with evidence that disturbances in the interactions between HS proteoglycans and growth factors/morphogens contribute to CNS pathology in various MPS types [4, 6, 10], it is likely that the binding of recombinant NK1 to the excess of extracellular and cell surface HS restores the physiological balance between HS proteoglycans, morphogens/growth factors, and receptors, thereby allowing proper receptor activation and subsequent signaling capable of reversing deregulated cellular processes in the MPS IIIB mouse brain.

Multiple studies have established that defective lysosomal activity causes dysregulation of autophagy, which is a relevant contributing factor to LSD pathology [2, 34–36]. Impairment of the autophagy-lysosomal pathway, critical for maintaining neuronal cellular homeostasis, drives neurodegenerative processes in several LSDs, including MPSs [2, 36, 45]. In our previous studies, we found that impaired lysosomal autophagic flux is linked to heart disease, valvular abnormalities, and cardiac failure in the MPS IIIB mouse model [46–48]. Additionally, a targeted metabolic analysis of *Naglu*^−/−^ mouse tissues revealed an imbalance in the metabolism of branched- chain amino acids (BCAAs), which are known to regulate lysosome biogenesis and autophagy mechanisms through the mechanistic target of rapamycin complex 1 (mTORC1) signaling [43]. In this study, we demonstrate that NK1 treatment of *Naglu*^−/−^ mice significantly reduces autophagosome accumulation in the neurons of the prefrontal cortex and hippocampus compared to untreated mice. The strong fluorescence intensity of the autophagosome marker protein [49] Lc3 observed in the neurons of the *Naglu*^−/−^ mouse brain aligns with recent data reported by Viana and colleagues [7], showing LC3 localization in the cytoplasmic puncta of cortical neurons in post-mortem samples from patients affected by neurological forms of MPS. In cellular models of MPS IIIB, we found that recombinant NK1 impacts autophagy pathways by downregulating the expression of autophagy-lysosome pathway (ALP) genes involved in autophagosome biogenesis [27]. The results of this study suggest that NK1 treatment may ameliorate the neurological phenotype of MPS IIIB by acting on mechanisms that regulate autophagy; however, further studies are needed to establish the precise molecular mechanisms through which NK1 affects autophagy in vivo.

Much evidence demonstrates that neuroinflammation and neurodegeneration are significant neuropathological phenotypes of the diseased brain in both MPS animal models and humans [12, 50–53]. In particular, astrocytosis and microglial activation have been shown to be present throughout the brains of MPS I, II, and III mouse models, including the MPS IIIB mouse model. Both activated microglia and activated astrocytes occur alongside the production of pro-inflammatory cytokines, whose levels are elevated in the brains of MPS murine models [11, 54–58]. In this study, treatment with recombinant NK1 protein resulted in a significant reduction of both astroglial and microglial activation in the cortex and hippocampus of *Naglu*^−/−^ mouse brains. Microglia and astrocytes are the predominant immune cells in the CNS, associated with innate immune responses and cytokine production in the brain [59]. They are also active regulators of neuronal synaptic interactions and neuronal plasticity, playing a major role in maintaining neuronal homeostasis and brain function [60]. Our findings suggest that NK1 treatment may reduce neuroinflammation, thereby restoring neuronal homeostasis and brain function in MPS IIIB disease.

Consistent with the aforementioned neuropathological findings, we also discovered that recombinant NK1 treatment significantly enhances motor activity and cognitive behavior in *Naglu*^−/−^ mice. Neurobehavioral abnormalities and cognitive decline represent the primary burden of MPS IIIB. Typically, behavioral issues such as hyperactivity and aggression manifest in the early stages of the disease, while the loss of motor skills is characteristic of its advanced stages. The rate of cognitive decline may vary among patients but consistently increases as the disease advances. Although the MPS IIIB mouse model is known to replicate the neurological phenotype of the human condition, discrepancies exist in the reported behavioral alterations in *Naglu*^−/−^ mice. In fact, some studies have shown MPS IIIB mice exhibiting hyperactivity in the open field test, while others reported reduced motor activity compared to control counterparts [10, 17, 18, 37]. However, in all instances, the mice became hypoactive as the disease progressed. Under our experimental conditions, at 32 weeks of age, *Naglu*^−/−^ mice were hypoactive, indicated by their decreased distance traveled in the open field and reduced time spent in the center compared to age-matched wild-type mice. Furthermore, the assessment of the discrimination index in object recognition, the number of alternations in the T-maze test, and the latency to fall in *Naglu*^−/−^ mice aligned with the prevailing consensus that MPS IIIB mice display impaired learning and memory abilities, thus mirroring the human disease. Interestingly, NK1 treatment enhanced both motor activity and cognitive skills in *Naglu*^−/−^ mice.

In conclusion, despite the limitations of this study, including the relatively small number of animals evaluated, the dose of recombinant NK1 administered, and the treatment duration, the results support our belief that NK1 administration could be beneficial for MPS IIIB patients and likely for other neurological MPS subtypes and other LSDs as well. This therapeutic approach offers the advantages of a low-risk safety profile, a reduced economic burden for manufacturing the recombinant protein, a route of administration that does not require invasive procedures but still requires hospitalization. Given the potential benefits for patients and the broader community, recombinant NK1 treatment for MPS IIIB disease warrants further development to advance toward future clinical trials.

## Materials and methods

### Mouse maintenance

Wild-type C57/BL6 mice were purchased from the Jackson Laboratory. *Naglu* knockout mice (*Naglu*^−/−^), originally developed by Prof. Elizabeth Neufeld at the University of California, Los Angeles, were created by inserting a neomycin resistance gene into exon 6 of the *Naglu* gene on the C57/BL6 background and were used in this study [17]. *Naglu*^−/−^ and Wt mice were genotyped as previously described [46]. For all experiments, adequate measures were taken to minimize any pain or discomfort. The mice included in the study were housed with no more than five per cage, maintained under identical conditions of temperature (21 ± 1 °C), humidity (60 ± 5%), and light/dark cycle (lights on from 6 am to 6 pm), and had free access to normal mouse chow and water. All mouse care and handling procedures were approved by the Institutional Animal Care and Use Committee (IACUC) of the Animal Facility of the Department of Molecular Medicine and Medical Biotechnology at the University of Naples Federico II (Naples, Italy) and authorized by the Italian Ministry of Health (Authorization n° 854/2021-PR). The experimental protocols were carried out in accordance with the principles and procedures outlined in the ARRIVE guidelines and EU Directive 2010/63/EU for animal experiments. Animals showing pre-existing illness or abnormal behavior were excluded based on predefined criteria.

### NK1 administration and animal groups

The recombinant NK1 fragment of HGF was produced using the yeast *Pichia pastoris* expression system and purified by heparin affinity chromatography as previously described [16, 61]. To investigate the neuroprotective effect of NK1 in the mouse model of MPS IIIB, *Naglu*^−/−^ and wild-type (Wt) mice of both sexes were treated starting at 8 weeks of age for 6 months with intraperitoneal injections (i.p.) of NK1 10 mg/kg/week. The other two groups of Wt and *Naglu*^−/−^ mice, respectively, received only the vehicle (PBS). The four study groups were as follows: vehicle-treated Wt (group 1, Wt vehicle) n. 10, 10 mg/kg NK1-treated Wt (group 2, Wt NK1) n. 9, vehicle-treated *Naglu*^−/−^ (group 3, *Naglu*^−/−^ vehicle) n. 9, and 10 mg/kg NK1-treated *Naglu*^−/−^ (group 4, *Naglu*^−/−^ NK1) n. 8. Animals were randomly assigned to experimental groups. Open field, object recognition, and T-maze spontaneous alternation tests were performed at 32 weeks of age. Brain sampling for histochemical analysis was carried out 1 week (7 days) after the last dose administration. Behavioral and histological analyses were conducted by investigators blinded to treatment groups.

### Immunohistochemistry and confocal microscopy

Immunohistochemistry and confocal microscopy procedures were performed as previously described [62]. Animals were anesthetized and transcardially perfused with saline solution containing 0.01 ml heparin (10 U/ml heparin in 0.1 M PBS), followed by 60 ml of 4% paraformaldehyde. Brains were rapidly extracted on ice, postfixed overnight at 4 °C, and cryoprotected in 30% sucrose in 0.1 M phosphate buffer (PB) containing 0.02% sodium azide for 24 h at 4 °C. The brains were then sectioned in a frozen state on a sliding cryostat at 40 μm thickness, in a rostrum-caudal direction. Free-floating serial sections were incubated with PB Triton X 0.3% and a blocking solution (0.5% milk, 10% FBS, 1% BSA) for 1 h and 30 min. The sections were incubated overnight at +4 °C with the following primary antibodies: anti-Lamp1 (mouse monoclonal anti-CD107a; 1:500; Sigma-Aldrich Cat# SAB4700416), anti-HS (mouse monoclonal anti-Heparan Sulfate (10E4); 1:500; AMSBIO Cat# 370255-1), anti-Lc3 (rabbit polyclonal antibody anti- Lc3; 1:500; Novus biologicals Cat# NB100-2331), anti-glial fibrillary acidic protein (rabbit polyclonal antibody anti-Gfap; 1:500; Abcam, Cambridge, UK, Cat# AB7260), and anti-ionized calcium binding adaptor molecule 1 (Iba1; rabbit polyclonal antibody; 1:500; Wako Diagnostic USA, Cat# 019–19741), and anti-NeuN (rabbit polyclonal antibody; 1:1000; Merck Millipore Cat# ABN78). Sections were incubated with the corresponding fluorescent-labeled secondary antibodies, Alexa 488/Alexa 594 conjugated anti-mouse/anti-rabbit IgGs (Jackson ImmunoResearch, Baltimore, PA). Nuclei were counterstained with Hoechst (Sigma-Aldrich, Milan, Italy). Images were captured using a Zeiss LSM700 META/laser scanning confocal microscope (Zeiss, Oberkochen, Germany). Images were taken with an optical thickness of 0.7 μm and a resolution of 1024 × 1024 [63].

### Fluorescence intensity analysis

The fluorescence intensity of Lamp1, HS, Lc3, Gfap, and Iba1 in tissue sections from the prefrontal cortex and hippocampus was quantified in terms of pixel intensity value using NIH image software, as previously described [64]. Briefly, digital images were captured with ×40 or ×20 objectives, applying identical laser power settings and exposure times across all photographs from each experimental set. Initially, images were thresholded to identify the positive signal; subsequently, the pixels expressing Lamp1, HS, Lc3, Gfap, and Iba1 were pinpointed. Finally, the number of pixels positive for antibodies was measured per microscope field. Images from the same regions of each brain area were compared. Results were expressed in arbitrary units. *n* = 3 mice per treatment group and 3 sections for each genotype.

### Delay-dependent one-trial object recognition task and Open field test

The behavioral tests used in this study to assess cognitive function were conducted in an arena measuring 50 × 50 × 40 cm. Mouse behavior was recorded by a camera positioned 2.5 m above the arena, and the data were analyzed using the tracking program EthoVision XT16 (Noldus). The object recognition task is based on the tendency of mice to explore a novel object versus a previously experienced object (familiar) when allowed to explore freely. In brief, two identical objects were placed in the arena, and the animals were permitted to explore for 10 min. Testing took place 24 h later in the same arena, where one of the original objects was replaced with a novel object, allowing the animals to explore for 5 min. Object exploration was measured by the time spent approaching an object (touching it with either mouse vibrissae, snout, or forepaws). The percentage of time spent exploring the novel object compared to the total time spent exploring both objects was used as a measure of object recognition: discrimination index = t novel/(t novel + t familiar) × 100 [65]. The open field test was carried out to assess locomotion, anxiety, and stereotypical behaviors, as previously described [65].

### T-maze spontaneous alternation

This procedure was conducted in an enclosed “T”- shaped maze (Med Associated, St. Albans, VT), where the long arm of the T (47 cm × 10 cm) serves as the start arm, and the short arms of the T (35 cm × 10 cm) function as the goal arms. In this task, the mouse was placed in the start arm, and after 5 seconds, the door was opened, allowing the mouse to choose and explore one of the goal arms. Once the mouse had fully entered the choice arm (with its tail tip completely inside), a guillotine door was closed, confining the mouse to the choice arm for 30 s. The mouse was then removed, the guillotine door was lifted, and the next trial was initiated. This procedure was repeated for a total of 10 trials. If the mouse did not make a choice within 2 min, the trial would end, and the next one would proceed. At the conclusion of each trial, the maze was cleaned of urine and feces [65].

### Statistics

Data were evaluated as means ± SEM. Statistically significant differences among the means were determined using one-way ANOVA followed by Tukey’s correction post-hoc test for fluorescence and behavioral tests. The variance was similar between the groups that were statistically compared. Statistical analyses were performed using GraphPad Prism 5.0 (La Jolla, CA, USA). The sample size was calculated using G*Power software to achieve 95% power, with an expected large effect size (0.60) and α = 0.05.

## Data Availability

Research data are stored in an institutional repository and will be shared upon reasonable request to the corresponding author.
